# Performance-based financing kick-starts motivational “feedback loop”: findings from a process evaluation in Mozambique

**DOI:** 10.1186/s12960-018-0320-x

**Published:** 2018-10-19

**Authors:** Jessica Gergen, Yogesh Rajkotia, Julia Lohmann, Nirmala Ravishankar

**Affiliations:** 1 ThinkWell, Rua da Azurara, Nr. 45, Bairro da Sommerschield, Maputo, Mozambique; 2 Heidelberg Institute of Global Health, Medical Faculty, Heidelberg University, Im Neuenheimer Feld 130.3, 69120 Heidelberg, Germany; 3 Nairobi, Kenya

## Abstract

**Background:**

Performance-based financing (PBF) reforms aim to directly influence health worker behavior through changes to institutional arrangements, accountability structures, and financial incentives based on performance. While there is still some debate about whether PBF influences extrinsic or intrinsic motivators, recent research finds that PBF affects both. Against this backdrop, our study presents findings from a process evaluation of a PBF program in Mozambique, exploring the perceived changes to both internal and external drivers of health worker motivation associated with PBF.

**Methods:**

We used a qualitative research design with in-depth, semi-structured interviews with health workers, which included a rank order exercise and focus group discussions. Interviews were analyzed by two researchers using thematic analysis techniques. Rank order frequency was calculated using weighted average methodology.

**Results:**

Health workers reported that PBF, overall, positively influenced their motivation by introducing or reinforcing both internal and external motivational drivers. Internal drivers included enhanced self-efficacy driven by goal orientation, healthy competition among colleagues, and job satisfaction. External drivers included an organized work environment, enhanced access to equipment and supplies, financial incentives, teamwork, and regular consultations with verifiers (a type of supervision). PBF stimulates an interactive relationship between internal and external motivational drivers, creating a feedback loop involving responsibility, achievement, and recognition, which increased perceived motivation.

**Conclusions:**

The PBF program helped workers feel that they had well-defined and achievable goals and that they received recognition from verification teams, management committees, and colleagues due to enhanced accountability and governance. Our paper shows that financial incentives could serve as the “driver” to kick-start the feedback loop, of responsibility, achievement, and recognition, in environments that lack other drivers. Understanding how PBF programs can be designed and refined to reinforce this feedback loop could be a powerful tool to further enhance and track positive motivational changes. For countries thinking about PBF, we recommend that policymakers assess the loop in their contexts, identify drivers, determine whether these drivers are sufficient, and consider PBF if they are not.

**Trial registration:**

We obtained ethical approval for the study protocol, data collection instruments, and informed consent forms from the Ethics Review Committee of the Centers for Disease Control and Prevention (CDC) [IRB 2015–190] and the Ethics Review Committee of the Mozambique Ministry of Health.

## Background

Health systems in low- and middle-income countries (LMICs) are plagued by several challenges, including weak management structures, inefficient information systems, and poor accountability for performance [1]. Health sector performance is critically dependent on health worker performance because service quality, as well as the accessibility and efficiency of health service delivery, is directly mediated by workers’ ability and willingness to apply themselves to their tasks [2]. Resource availability and worker competence are essential but not sufficient to ensure desired worker performance [3]. Hence, motivation—which we define as an individual’s degree of willingness to exert and maintain effort toward organizational goals [2]—is critical for ensuring that qualified workers perform well. Against this backdrop, performance-based financing (PBF) has emerged as a promising intervention for improving the delivery of high-quality healthcare services by enhancing health worker motivation.

A handful of studies have investigated whether PBF does indeed enhance health worker motivation, with mixed results. In Nigeria [4], Burundi [5], Tanzania [6, 7], and Sierra Leone [8], PBF had an overall positive effect on motivation, whereas in Benin [9], DRC [10], Pakistan [11], and Zambia [12], PBF did not appear to change levels of health worker motivation. In Afghanistan, PBF was negatively associated with motivational factors, although it had a significant positive effect on quality of care [13].

Motivation is often conceptualized using an intrinsic-extrinsic dichotomy, where behavior is categorized as extrinsically or intrinsically motivated based on whether or not it was induced by some external stimulus [14, 15]. PBF as an intervention—particularly the individual financial incentives, which are part of most PBF schemes—is believed to primarily enhance extrinsic motivation. However, elements of PBF may foster both extrinsic and intrinsic motivation. Evidence on the motivational mechanisms of PBF remains scarce, but some studies have found that PBF can stimulate intrinsic forms of motivation, such as transforming the work environment [4, 9, 12, 16, 17], giving health workers opportunities to improve skills, enhancing recognition from the community [4, 19], and strengthening professional pride and responsibility [9, 19]. It thus appears that PBF as a complex systems intervention has the potential to influence many different types of motivational determinants [20–22].

This study explores the different ways PBF influences health worker motivation in the context of a PBF pilot in Mozambique. Our objective is to assess the perceived changes, by health workers, on motivational drivers associated with the introduction of PBF.

### Study setting

The CDC PEPFAR program in Mozambique financed the implementation of a PBF program targeting government-owned health facilities in two provinces of Mozambique (Gaza and Nampula) in early 2011. By 2015, the PBF program included 138 health facilities (73 facilities in Gaza and 65 facilities in Nampula), but was ended in 2016 and never scaled past these two provinces.

The program focuses on providing HIV-related services, primarily for pregnant women and children. Twenty-one incentivized indicators are clustered in four groups by the type of service: prevention of mother-to-child transmission (PMTCT), pediatric HIV, adult HIV/TB, and maternal and child health (MCH). Payment is based on the sum of three components: quantity, quality, and equity (for rural and hard-to-reach areas). Health facilities report on PBF indicators monthly and submit aggregated reports on a quarterly basis. Data verification and payment cycles occur every quarter and are conducted jointly by the implementing NGO and the Provincial Health Office (DPS). PBF earnings are re-invested in the facility (40%) and used to provide salary top-ups to staff (60%). Salary top-ups are distributed among all health facility staff based on such pre-determined criteria as years of experience, facility tenure, level of education, and professional position. According to internal program data, salary top-ups account for between 20 and 50% of an average health worker’s salary, and facility investments for approximately 50% of total facility operating costs.

## Methods

### Data collection and analysis

To understand perceptions of health worker motivation, we used a qualitative research design with in-depth, semi-structured interviews, including a rank order exercise and focus group discussions. Our sample included 56 health workers and administrators (Table 1). Motivational determinants were identified through a free-listing exercise where health workers were first asked to define motivation and then asked to write a list of all the determinants or drivers of motivation associated with their jobs in June 2015. They were then asked to describe changes they observed within the health facility since the introduction of PBF and to reflect on the positive, negative, or null changes related to PBF across 15 motivational determinants (Table 2).

**Table 1:** Profile of study participants (worker motivation)

	Gaza	Nampula	Total
	*N* (% of province)	*N* (% of province)	*N* (% of total)
Total participants			
In-depth interviews	16 (84%)	17 (85%)	33 (85%)
Focus group discussions	3 FGDs (16%) with an avg. of 4.6 participants	3 FGDs (15%) with an avg. of 3.3 participants	6 (15%) with a total of 24 participants
Total	19 (100%)	20 (100%)	39 (100%)
Sex, no. (%)			
Male	8 (47%)	6 (35%)	14 (41%)
Female	9 (53%)	11 (65%)	20 (59%)
Total	17 (100%)	17 (100%)	34 (100%)
Professional position, no. (%)			
Nurse (chief/head)	0 (0%)	2 (12%)	2 (6%)
Nurse (general)	1 (6%)	1 (6%)	2 (3%)
Nurse (MNCH)	5 (29%)	6 (35%)	11 (32%)
Medical technician (general)	7 (41%)	8 (47%)	15 (44%)
Counselor	2 (12%)	0 (%)	2 (6%)
Facility administrator/manager	2 (12%)	0 (%)	2 (6%)
Total	17 (100%)	17 (100%)	34 (100%)
PBF exposure (avg. no. of quarters)	9.3 Q (2.3 years)	11.7 Q (2.92 years)	10.6 Q (2.65 years)
Number of health facilities	8	9	17
Facility level, no. (%)			
Tertiary	2 (25%)	2 (22%)	4 (25%)
Primary	6 (75%)	7 (78%)	13 (75%)
Total	8 (100%)	9 (100%)	17 (100%)

*Q* quarter (equivalent of 3 months)

**Table 2:** Perceived motivational determinants ranking (rank order) by health workers

Motivational determinants/drivers	Average weighted rank
More organized work environment^a^	3.65
Ability to do a good job (perform well)	3.42
Bonus or salary top-up	3.31
Competitive pressure^b^	3.11
Teamwork	3.10
Satisfaction with personal performance	3.0
Better access to equipment and infrastructure	2.93
Regular consultations with supervisors	2.72
Prestige among the community	2.41
More training opportunities	2.57
Feeling proud as a professional^c^	2.0
Not influenced by PBF	
Advancement in knowledge to complete work	
Meaningfulness/purpose of my work tasks	
Trust from patients/clients (politeness/relationships with patients)	
Opportunity for professional advancement	

^a^“More organized work environment” and “better access to equipment and infrastructure” were grouped together due to similarity in description

^b^“Pressure to perform” and “teamwork” are discussed together due to linkages in how teamwork dynamics were influenced by pressure or competitiveness

^c^“Feeling proud as a professional” included “prestige within the community” as one source of professional pride

During in-depth interviews and focus groups, clinic-focused health workers completed an exercise with the 15 determinants, ranking them from the most to least, or not at all, influenced by PBF. Data were collected, between November and December 2015, at 17 public (government-owned and government-operated) health facilities in Nampula (nine) and Gaza (eight), 76% of them primary health centers and 71% classified as rural or peri-urban. We selected a range of facilities based on size, geographic location (rural, peri-urban, urban), and facility performance data. Each facility had participated in the PBF program for a minimum of 18 months.

All interviews were conducted by two researchers and transcribed in Portuguese, then imported into ATLAS.ti version 1.0.14 for thematic analysis. Two or three researchers conducted focus group discussions in three urban (two Gaza, one Nampula) and three rural (one Gaza, two Nampula) health facilities. Two researchers coded interviews line-by-line and analyzed them to identify recurring themes and variations across responses. Initial themes and sub-codes were organized based on the theoretical model of intrinsic and extrinsic dichotomy, although interactions and relationships between domains were further noted through memos and subsequent coding. Differences among health worker cadres, provinces, and health facility level were all assessed based on coding patterns and quotations for each dimension. Rank order frequency was calculated using weighted average methodology for determinants most influenced by PBF.

## Results

Health workers reported that PBF had influenced motivation in direct and indirect ways, with 11 motivational determinants ranked as influenced by PBF (Table 2). Four determinants were grouped together during the analytical process due to similarity in description and conceptualization by informants (see notes below table). Four determinants were identified as not influenced by PBF. There were no notable differences between the responses or rankings in the two different provinces.

Table 3 contains illustrative quotations (annex).

**Table 3:** Illustrative quotations by motivational domains

Motivational domains	Key quotations
Work environment	“We had a more organized work environment, because of the [new] chairs, lockers, the fans ... the environment became more comfortable and safer for my patients.” IDI55_Nampula “I was not satisfied working in a place that is not clean. [Implementing] PBF brought hygiene supplies and cleaning. [Now] my work environment is beautiful and well organized. I’m happy with the environment, so I’m satisfied with my work too, because I work well when I’m in a good place.” IDI34_Nampula “I will say that the more organized the work environment, the better our activities comply with our business and management plans and our work environment goals. Because of PBF, we made plans to ensure our workplace focuses on patient care. We planned what equipment was needed. All this contributes to a healthy work environment. Before, we did not have a fan to at least reduce the heat in our office, but now allocating [for equipment] through the PBF means a positive work environment where the professionals and patients do not feel suffocated from the heat.” IDI25_Gaza
Equipment and infrastructure	“It would take 15 years for each facility [to upgrade] if we did not have the PBF [to supply] the porch, these comfortable chairs, upgraded bathrooms, and electrification. We know a district budget is never enough to meet all our needs. The PBF therefore has great impact … because it improves working conditions for health professionals.” IDI35_Nampula “For example, we have improved chairs; before we were not comfortable. Now I will not tire so easily [because] I am well seated and can continue with my work without discomfort. Also they have given us fans [for the heat] and the ventilation helps me spend more time in a small office and meet the patients without discomfort, [improving] my performance.” IDI18_Gaza
Bonuses	“It all depends on the patients that meet (come) to our health facility because the amount is determined by the data, if the number of patients is greater than we gain more ... the biggest piece of the cake is the payment that EGPAF provides, so I feel like we have improved because we try to achieve more to earn more...we make ourselves available of those patients who feel the suffering, and we treat them as our friends, so that they never want to leave to go to another health facility. If patients left that would be at the expense of the bonus we receive from EGPAF, and would also not help us achieve our health goals.” IDI31_Nampula “Only some providers contribute to the performance that is counted toward the payment. We do not perform the same jobs [even if] we have the same [level of] performance, only some of that performance counts to increase the payment. So this is a challenge. Because those who are involved with PBF services have their performance measured and have to watch over the situation. All of us always tend to work hard for the bonus because it ends up benefiting the overall performance of the health facility and helps the others who count to perform better.” IDI31_Nampula “We are worried; why this difference [in bonuses] if the payment is based on performance? It [should not be] based on the career or the position that the person has. That is why there were personal contracts with the PBF ... that each individual had to sign. So the only thing I personally criticize is [the inequity] in bonuses because it should be the same for everyone.” IDI43_Nampula “We have so many employees and only cover some of the PBF services so the bonuses are very small for me. Sometimes I do not notice them as being different from my regular salary.” IDI07_Gaza
Teamwork	“I think yes, there was a change, because [now] we always talk about the instruments and indicators and other PBF matters... [showing] our strong commitment.” IDI04_Gaza “We say to colleagues that the PBF is for everyone. So all of us have to make every effort together to achieve those goals or to have those indicators to avail ourselves of [the benefits of] PBF. So there is a concerted effort to achieve these goals.” IDI21_Gaza “I think the individual [perspective] ends up being part of a collective. We are earn more as a collective so we work together to improve. Because first comes motivation, and then when people are motivated [they] end up doing things to improve the quality of care. And when we improve the quality of care [we] also improve … the achievement level [of staff in working toward] the objectives of each health sector to improve indicators.” IDI14_Nampula
Regular consultations with supervisors	“Before PBF, if we did not do our job, accountability was not in place, so no one said whether it was done correctly or done at all.” IDI055_Nampula “It has been pretty positive to work with the verification team because there is always interaction between the evaluator and the employee … to show how much we have accomplished, how much we should have done, and why we did not reach our goals. And then we try to work to overcome what we failed in before over the next few months.” IDI15_Gaza “The [new approach to] evaluation is positive because before PBF we worked to produce information to report to the provincial directors and then to the Ministry. And this information somehow was charged as we achieved certain goals, and we worked accordingly. But with PBF…we make more effort to reach the goals we are given because at the end of a certain period we will receive a bonus for that [extra] effort and it will be acknowledged. With the start of PBF we felt like, efforts were duplicated because in some ways our efforts were to improve our performance both for government goals and PBF goals- sometimes the same. Then after some time in PBF, it was really positive because we doubled [our efforts] knowing we were going to receive something in return.” IDI21_Gaza (Manager)
Application of knowledge and skills to deliver a good job	“In terms of quality, especially in the provision of services ... we did our duty ... to see the patients and deliver treatment. But when PBF began it provided an incentive to succeed. The more important outcome of the incentives is the quality of service provision, so we look at the standards and ask for instructions, and we are committed to doing a good job. This is a significant change, because before we came to do our duties and then went home. But now my unit is doing a better job and I stay my whole shift time or even past my shift time make sure each patient is seen and provided care.” IDI51_Nampula “We applied the knowledge from verifiers. When they come to present to us PBF and how to get more for our work. PBF taught us some of the things to increases on time with patients. But no, we have not looked at any new clinical trainings from the DPS (district health manager).” IDI06_Gaza
Competition among my colleagues	“We compare our performance among ourselves. We track activities and so does the medical chief. No my colleagues will show up to say I will do some tests (HIV) because you did your part. This ends up influencing the indicators and our performance, and we do it together but comparing with each other. Staff more clearly know what they need to do ... to improve performance, and then together we share the incentive.” IDI60_Nampula “We all share a goal now, which will be reached this quarter. If the goal is high, the bonus is also high, and then it helps a lot to motivate the whole team, or the institution, as well as their own health officials to seek more, run more, go go go. And we encourage each other when we see one person not doing their part.” IDI17_Gaza
Feeling satisfied with the work I complete	“I am satisfied with my work for the moment because I know what work I have to do, and at the end of the quarter there will be bonuses. I have more motivation because the PBF [has helped] my work to be fully organized in my office, and my colleagues are also organized for their patients. So PBF really makes me very satisfied with my own work.” IDI35_Nampula “We had to achieve the goals, and to achieve those goals and be noticed within the Health Unit, we had to work hard, we had to follow up with the patients. This drive is satisfying for me in this job.” IDI25_Gaza “Patients will notice the changes at the facility, the fresh paint and less trash … and are also happy to have reduced wait times. They tell me we are doing a better job and that is why PBF has improved my satisfaction.” IDI04_Gaza
Prestige within the community	“I think EGPAF wants to improve the indicators as a way to help us as employees, the community, the hospital, and the Directorate, because they offer a bonus depending on how well we work. That bonus helps us help the community and the hospital. To us, it means kind, humanized care … encouraging us to work more for the Health Center to improve infrastructure and everything else. For the community, we see that the more people are coming and feel comfortable during their wait” IDI36_Nampula “People were aware that we have to be paid decently for services (provided) because if we do not, we cannot provide as much service, and patients will abandon us. And if we abandon our indicators in vain, then we cannot succeed. It is true that it is not right either to come (to the health facility) with a bad mood, but this is normal happens to everyone, but in terms of performance and delay with the wait time- we are all now aware, if we do not offer high-quality services ... to our users, they abandon us. What will happened to us with PBF indicators if we do not provide good service is no increased performance.” IDI51_Nampula
Feeling proud as a professional	“Improving services ... is a way of motivating employees. If the employee feels motivated soon he or she will ... work with more desire for achieving quality. ... if yesterday had two patient and motivated professional can make even greater efforts to have MORE patient because it has something that motivates you ... she has benefit, yes, then it is in this context that I was saying that ... improves the quality of work and not only ... the very PBF as I was saying ... quality work also with the same value that we have at the clinic we can buy some equipment that before we did not have the facility, then more so the work equipment and also improves ... is quality work ... yes.” IDI34_Nampula “As a health professional, I feel proud … of the work that I do. For example, I can follow a mother from the ANC visit until delivery, then follow the same mother and child. This leaves me satisfied that I gave a good follow-up and it was worth it.” IDI24_Gaza

## Key motivational determinants

### More organized work environment

Health workers described PBF as a tool for creating a more organized work environment. Medical and office supplies required for day-to-day work were more readily available, enhancing efficiency and comfort. Workers felt that PBF had changed their typical workflow, by organizing the environment and patient books, increasing coordination of which patients they should see or follow up.

There were several notable structural changes that health workers reported. The physical appearance of the facilities, including wall painting, infrastructure upgrades (e.g., latrines and septic tanks), and cleanliness (less trash, more waste bins available, clean consultation rooms), was most frequently reported. For health facilities with “deteriorating infrastructure and inadequate equipment and supplies,” PBF transformed the facility with “newly painted walls with functional windows and doors and general cleanliness.” These structural improvements influenced workers’ pride in and satisfaction with the appearance of their facilities.

Respondents further described how PBF introduced specific, timely, and frequent verification processes, whereby provincial project staff and DPS would visit each facility on a consistent and planned basis, and verify the reported data through register checks. Facilities reacted by instituting daily and weekly record-keeping and self-verification to ensure the completeness and quality of the data. Management committee meetings were conducted monthly to plan and discuss PBF.

### Ability to perform well

Health workers reported being more committed to their work, giving up other personal and professional duties to ensure they completed tasks and increased the success of PBF in their facilities. Doing a “good job” was perceived as following instructions, completing the registry, and communicating with team members. They also linked doing a good job to adherence to clinical protocols; while health workers reported having access to and understanding correct clinical protocols even before PBF, the scheme increased attention to the protocols as well as accountability for adherence. In general, all respondents suggested that their colleagues were as committed to and proud of the work as they were themselves.

### Bonus or salary top-up

Although respondents did not rank performance bonuses as the most significant aspect of PBF that enhanced their motivation, every health worker interviewed described the effects of incentives in their work. The financial bonus made the health workers feel more appreciated and respected. Workers felt that the bonus made it worth completing their work, despite challenges. Payments were timely, and some facilities made efforts to increase transparency of incentives by posting what everyone received on the health facility bulletin.

While the salary top-ups were widely appreciated, some workers felt that the distribution of funds was unfair. They questioned the higher bonuses that technicians and physicians received, even though lower-level cadres such as nurses and lay counselors exerted greater effort. Most nurses interviewed argued that since everyone was working together to maximize payments, they should all benefit equally. Further, for health workers at larger facilities, the bonuses were reportedly too small to dramatically influence behavior or motivation. According to program data, staff bonuses represented 15% to 25% of the monthly salary for higher-level health staff (e.g., doctors) or for those working in larger facilities and 30% to 50% of the monthly salary for lower-level staff (e.g., activists, janitorial technicians) or for rural staff in small facilities.

### Constructive competition and teamwork

Respondents reported that PBF had introduced healthy competition among colleagues. Health workers were more conscious of the work completed by colleagues, which inspired each to work hard to earn their “piece of the pie.” PBF created this sense of competition by refocusing on performance, enhancing transparency, and increasing accountability.

Management boards were formed as a part of the PBF program for facility fiscal planning, but often included only facility administrators (e.g., head nurses and senior medical technicians). Workers felt that they were “left out of important decisions for the facility” because the management boards were neither participatory nor inclusive of their opinions and concerns. In contrast, the fact that everyone received a bonus meant that all health workers were more motivated to help each other with clinical tasks. Most noticed increases in the frequency of communication with other workers assigned to different services as well as meetings with the DPS and the implementing NGO’s teams.

### Satisfaction with personal performance

Health workers felt that while PBF resulted in a heavier workload, it also enhanced their drive to complete more of their tasks, which led to a feeling of job satisfaction. Respondents agreed that workflow and managerial organization had improved, which enhanced workers’ ability to finish clinical tasks. Receiving recognition from verifiers or DPS supervisors led workers to report feeling greater job satisfaction.

### Regular consultations with supervisors

PBF did not directly affect the physical presence of district supervisors or use of supportive supervision techniques. However, health workers said that the frequency and consistency of visits by verification teams were positive changes. The workers felt constantly monitored but were happy about the “supportive” nature of the supervision they received from the verification team.

## Interactive effects: PBF feedback loop

**Fig. 1:**
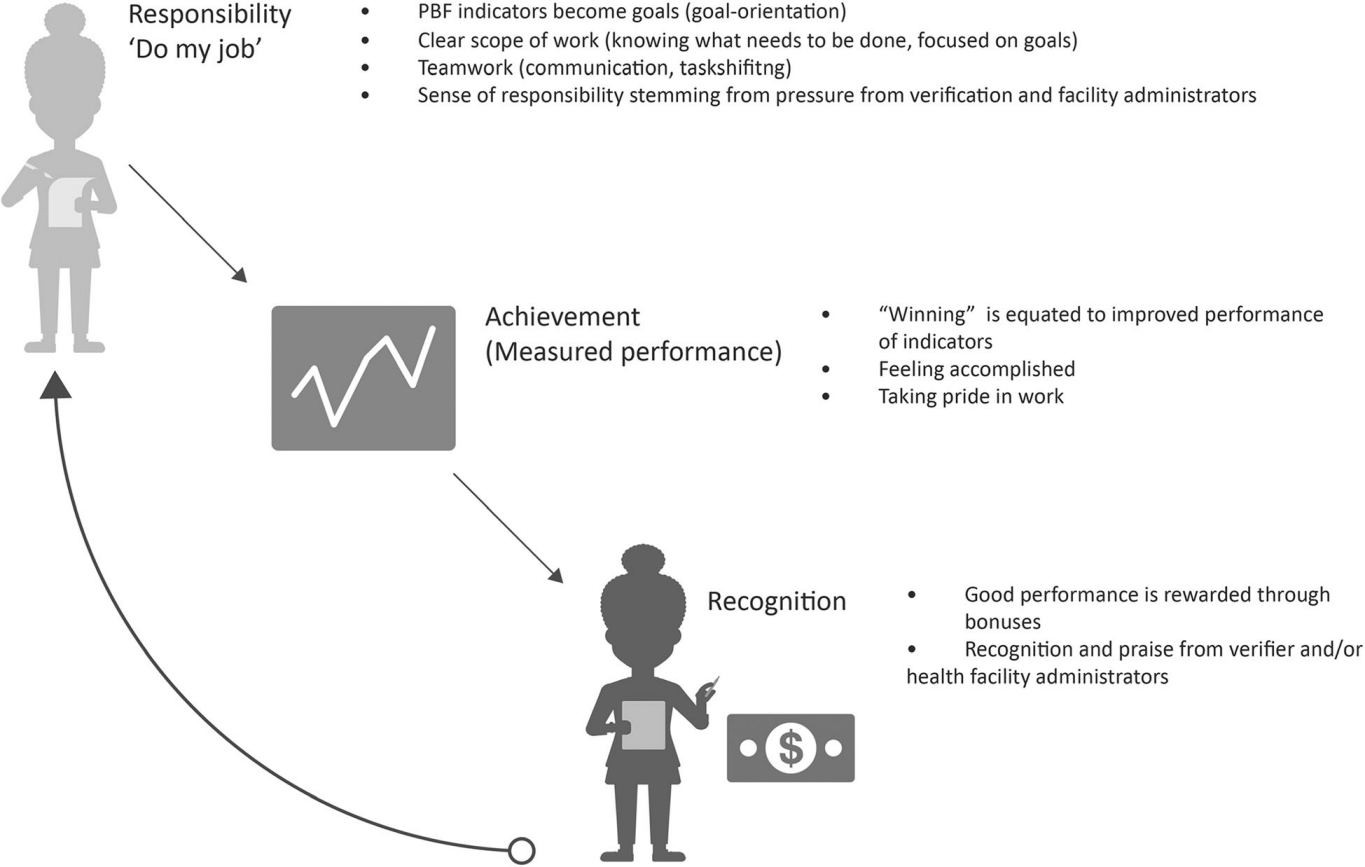
PBF’s feedback loop to enhance health worker motivation. The figure depicts the derived feedback loop between the processes that are strengthened by PBF

Health workers often described how the changes in motivational drivers to PBF were interactive. External changes triggered other changes in their intrapersonal motivational drivers and vice versa. Figure 1 displays this motivational feedback loop created by PBF’s institutional and organizational components.

Over two thirds of workers believed PBF had resulted in a shift in orientation toward clearly defined goals. Workers were better able to identify the facility’s performance goals, feel responsible for these goals, and focus their efforts on “doing their job.” Moreover, sharing the same goals inspired a feeling of camaraderie throughout the facility.

Respondents linked clarity of goals with feelings of ownership and efficacy, which ultimately resulted in greater motivation. They credited frequent verification for engendering greater accountability around individual and facility performance. They also reported feeling more motivated to achieve shared goals because they could do their jobs more effectively, given changes in the work environment. In their view, PBF had improved facility infrastructure, staff relationships, and workflow.

### Achievement

Many health workers described how PBF instilled in them a new sense of accomplishment and professional pride, which they viewed as highly motivating. This new sense of achievement was brought about by introducing and institutionalizing performance monitoring and managerial processes, or “regimenting” of the health facility. Respondents viewed this as a crucial driver of performance and of heightened feelings of motivation and job satisfaction.

### Recognition

The achievement of well-defined goals resulted in rewards, both financial and non-financial. Good performance led to increased bonuses for workers, as well as greater worker investment in the health facility. Most workers were very happy to receive additional payments and reported that it boosted morale and allowed them to take care of their families. These bonuses motivated them to work harder (longer hours, lower absenteeism) and communicate more frequently with other clinical team members. The reward itself garnered recognition from family and close friends.

Beyond the payments, workers also reported feeling recognized for their work through the PBF program’s measurement and verification of results. Health workers described how the accountability and governance mechanisms institutionalized in the context of PBF allowed for more praise and positive reinforcement from verification teams, management committees, and the community.

## Discussion

PBF is a policy reform that aims to directly influence health worker behavior through several mechanisms, including changes to institutional arrangements, accountability structures, and financial incentives based on performance [20, 23–25]. The increasing use of PBF demands a more in-depth and comprehensive understanding of how PBF influences health worker motivation and behavior [25–27]. Research on PBF indicates that some PBF schemes do have the capacity to enhance overall motivation for providing PBF-targeted services [18, 28–31]. The effect of PBF on motivation is usually explained as a stimulus or extrinsic driver of motivation [32]. The literature documenting PBF’s effects on health worker motivation, however, suggests that this conceptualization might be too narrow to adequately capture the complex motivational effects of PBF [12, 18, 33, 34].

In line with this body of literature, our results show that health workers perceived both external changes in the work environment and changes within themselves that enhanced or hindered feelings of motivation. Health workers described how the PBF program has significantly improved the organization of the facility and the availability of equipment and supplies required for their day-to-day job activities, which they found to be motivating. Results from Nigeria and Benin captured a similar relationship between health worker motivation and working conditions [27, 35]. PBF program designers and implementers should note that the structural changes and re-investment in the facility can motivate staff and make it easier for them to do their jobs.

Workers also shared a positive perception of incentives, supported by the sense of accomplishment and satisfaction of earning an additional reward. The aspiration for additional income or consistent improvement of health facilities motivated staff to provide patient care aligned with PBF targets or what they referred to as goals [32]. In some cases, distribution of incentives created feelings of unfairness because certain cadres or workers with heavier patient loads or administrative duties felt that they should receive a higher proportion of the bonus compared to facility staff who do not work on PBF services. Similarly, in larger facilities with many staff, the incentives were very low and subsequently were not a significant source of enhanced motivation.

Existing PBF schemes differ considerably in terms of both the share of PBF payment that is reserved for salary bonuses (versus investment in the facility) and how the bonuses are divided between staff. One review of 20 PBF schemes showed that staff bonuses as a share of total PBF payments varied between 25 and 80%, and documented a variety of formulas for distributing payments among workers within a facility [30]. Given the evidence that incentives can be a motivational driver in PBF, more research is required to understand how PBF payment formulas and allocative disbursements between facility investment and individuals can drive both motivation and overall health system strengthening.

The broader health system literature shows that internal motivational drivers are equally important in shaping health worker motivation [18]. In Mozambique, health workers reported changes in improved teamwork with colleagues, pride and satisfaction associated with completing their work, and appreciation from verifiers and colleagues. In other LMICs, internal motives, a sense of responsibility, pride in one’s work, and recognition from the community have been shown to be key motivational drivers [36–38]. From the health worker’s perspective, the changes contributing to such enhanced feelings included strengthened management structures, primarily through the verification process and by facility administrators checking on service delivery and the register [39, 40].

Importantly, our findings found that PBF strengthens an existing feedback loop, exhibited most clearly when providers described changes to internal and external drivers together and in a specific cascade. One of the most common interactive descriptions involved financial incentives that resulted in other external and internal shifts in motivation. Financial incentives were perceived as highly influential and reportedly increased health workers’ motivation to exert additional effort to meet increased demand, feel pride in one’s work, and communicate with one another about service delivery. Further, when questioned about how PBF creates and sustains changes in perceived motivation, a feedback loop involving responsibility, achievement, and recognition. The institutional and organizational arrangements that PBF changed helped facilitate this cycle or feedback loop and helped workers feel that they had well-defined, achievable goals to work toward, while accountability and governance meant that many health workers received praise from verification teams, management committees, and colleagues.

Although this interactive relationship has not been made explicit in PBF publications, feedback loops are an integral component of all systems and evidence of this feedback loop is supported by evidence. For instance, in Zambia, PBF enhanced job satisfaction through better working conditions and more effective supervision, in addition to higher job satisfaction with compensation, feelings of accomplishment, and financial autonomy [12]. In Malawi, health workers were motivated by the goals and direction introduced by PBF [18]. A more enabling working environment induced by PBF (e.g., improved resource situation, enhanced supervision, better management support) (1) allowed health workers to improve their performance by aiming for these goals, (2) created new feelings of self-efficacy and pride as health workers recognized their accomplishments, and (3) were positively reinforced by managers, the community, and implementers, verbally and through PBF rewards. This fueled a positive spiral of motivation to continuously improve performance [18].

### Limitations

The methodology adopted in this study—capturing information on the effects of PBF health systems through semi-structured interviews with health workers—has several limitations. First, respondents were selected in collaboration with facility management, which could have led to a biased sample of motivated or positive health workers. Although the evaluation was conducted by a third-party evaluator, project implementers and occasionally DPS accompanied our data collectors to the facility to facilitate the introduction and coordination of the interviews; their presence could have influenced the views expressed by study participants. The interview guide was open-ended, but all health workers were probed on personal motivational changes attributed to PBF. The motivational determinants were defined through free-list exercises with health workers not included in the study but working in the same provinces as comparable health facilities prior to the data collection period. The probing may have introduced concepts that the workers would not have mentioned or noted on their own.

## Conclusion

Our study found that PBF led to changes in both internal and external drivers of health worker motivation and created an interactive feedback loop involving responsibility, achievement, and recognition. The institutional and organization arrangements that PBF created allowed for workers to feel that they had well-defined goals and could achieve these targets, and accountability and governance allowed for many health workers to receive praise from verification teams, management committees, and colleagues.

Our paper shows that financial incentives can serve as the “driver” to kick-start the loop in environments that lack other drivers. For countries thinking about PBF, we recommend that policymakers assess the loop in their contexts, identify drivers, determine whether these drivers are sufficient, and consider PBF if they are not.

## References

[1] Mills A (2014). Health care systems in low- and middle-income countries. N Engl J Med.

[2] Franco LM, Bennett S, Kanfer R (2002). Health sector reform and public sector health worker motivation: a conceptual framework. Soc Sci Med.

[3] Scott A, Sivey P, Ait Ouakrim D, Willenberg L, Naccarella L, Furler J (2011). The effect of financial incentives on the quality of health care provided by primary care physicians. Cochrane Database Syst Rev.

[4] Bhatnagar A, George AS (2016). Motivating health workers up to a limit: partial effects of performance-based financing on working environments in Nigeria. Health Policy Plan.

[5] Bertone MP, Meessen B (2013). Studying the link between institutions and health system performance: a framework and an illustration with the analysis of two performance-based financing schemes in Burundi. Health Policy Plan.

[6] Chimhutu V, Lindkvist I, Lange S (2014). When incentives work too well: locally implemented pay for performance (P4P) and adverse sanctions towards home birth in Tanzania—a qualitative study. BMC Health Serv Res.

[7] Chimhutu V, Tjomsland M, Songstad NG, Mrisho M, Moland KM (2015). Introducing payment for performance in the health sector of Tanzania—the policy process. Glob Health.

[8] Bertone MP, Lagarde M, Witter S (2016). Performance-based financing in the context of the complex remuneration of health workers: findings from a mixed-method study in rural Sierra Leone. BMC Health Serv Res.

[9] Paul E, Sossouhounto N, Eclou DS (2014). Local stakeholders’ perceptions about the introduction of performance-based financing in Benin: a case study in two health districts. Int J Health Policy Manag.

[10] Fox S, Witter S, Wylde E, Mafuta E, Lievens T (2014). Paying health workers for performance in a fragmented, fragile state: reflections from Katanga Province, Democratic Republic of Congo. Health Policy Plan.

[11] Witter S, Zulfiqur T, Javeed S, Khan A, Bari A (2011). Paying health workers for performance in Battagram district. Pakistan Hum Resour Health.

[12] Shen GC, Nguyen HT, Das A, Sachingongu N, Chansa C, Qamruddin J (2017). Incentives to change: effects of performance-based financing on health workers in Zambia. Hum Resour Health.

[13] Dale E (2014). Performance-based payments, provider motivation and quality of care in Afghanistan.

[14] DeCharms R (1958). A self-scored projective measure of achievement and affiliation motivation. J Consult Psychol.

[15] DeCharms R (1968). Personal causation: the internal affective determinants of behavior.

[16] Kalk A, Paul FA, Grabosch E (2010). ‘Paying for performance’ in Rwanda: does it pay off?. Tropical Med Int Health.

[17] Paul E, Renmans D (2017). Performance-based financing in the heath sector in low- and middle-income countries: is there anything whereof it may be said, see, this is new?. Int J health Plann manage.

[18] Lohmann Julia, Wilhelm Danielle, Kambala Christabel, Brenner Stephan, Muula Adamson S, De Allegri Manuela (2017). ‘The money can be a motivator, to me a little, but mostly PBF just helps me to do better in my job.’ An exploration of the motivational mechanisms of performance-based financing for health workers in Malawi. Health Policy and Planning.

[19] Aninanya GA, Howard N, Williams JE, Apam B, Prytherch H, Loukanova S (2016). Can performance-based incentives improve motivation of nurses and midwives in primary facilities in northern Ghana? A quasi-experimental study. Glob Health Action.

[20] Meessen B, Shroff ZC, Ir P, Bigdeli M. From scheme to system (part 1): notes on conceptual and methodological innovations in the multicountry research program on scaling up results-based financing in health systems. Health Systems & Reform 2017. 10.1080/23288604.2017.1303561 31514678

[21] Shroff Zubin Cyrus, Bigdeli Maryam, Meessen Bruno (2017). From Scheme to System (Part 2): Findings from Ten Countries on the Policy Evolution of Results-Based Financing in Health Systems. Health Systems & Reform.

[22] Witter S, Toonen J, Meessen B, Kagubare J, Fritsche G, Vaughan K (2013). Performance-based financing as a health system reform: mapping the key dimensions for monitoring and evaluation. BMC Health Serv Res.

[23] Kinfu Y, Dal Poz MR, Mercer H, Evans DB (2009). The health worker shortage in Africa: are enough physicians and nurses being trained?. Bull World Health Organ.

[24] Nimpagaritse M, Korachais C, Roberfroid D, Kolsteren P, Zine Eddine El Idrissi MD, Meessen B (2016). Measuring and understanding the effects of a performance based financing scheme applied to nutrition services in Burundi-a mixed method impact evaluation design. Int J Equity Health.

[25] Renmans D, Holvoet N, Criel B, Meessen B (2017). Performance-based financing: the same is different. Health Policy Plan.

[26] Renmans D, Holvoet N, Orach CG, Criel B (2016). Opening the ‘black box’ of performance-based financing in low- and lower middle-income countries: a review of the literature. Health Policy Plan.

[27] Lohmann J, Souares A, Tiendrebéogo J, Houlfort N, Robyn PJ, Somda SMA (2017). Measuring health workers’ motivation composition: validation of a scale based on self-determination theory in Burkina Faso. Hum Resour Health.

[28] Ssengooba F, McPake B, Palmer N (2012). Why performance-based contracting failed in Uganda--an “open-box” evaluation of a complex health system intervention. Soc Sci Med.

[29] Witter S, Fretheim A, Kessy FL, Lindahl AK (2012). Paying for performance to improve the delivery of health interventions in low- and middle-income countries. Cochrane Database Syst Rev.

[30] Gergen J, Josephson E, Coe M, Ski S, Madhavan S, Bauhoff S (2017). Quality of care in performance-based financing: how it is incorporated in 32 programs across 28 countries. Glob Health Sci Pract.

[31] Josephson E, Gergen J, Coe M, Ski S, Madhavan S, Bauhoff S (2017). How do performance-based financing programmes measure quality of care? A descriptive analysis of 68 quality checklists from 28 low- and middle-income countries. Health Policy Plan.

[32] William Savedoff SP (2010). Basic economics of results-based financing in health.

[33] Lannes L, Meessen B, Soucat A, Basinga P (2015). Can performance-based financing help reaching the poor with maternal and child health services? The experience of rural Rwanda. Int J Health Plann Manag.

[34] Brenner S, Wilhelm D, Lohmann J, Kambala C, Chinkhumba J, Muula AS (2017). Implementation research to improve quality of maternal and newborn health care, Malawi. Bull World Health Organ.

[35] Bonfrer I, Soeters R, Van de Poel E, Basenya O, Longin G, van de Looij F (2014). Introduction of performance-based financing in Burundi was associated with improvements in care and quality. Health Aff (Millwood).

[36] Mathauer I, Imhoff I (2006). Health worker motivation in Africa: the role of non-financial incentives and human resource management tools. Hum Resour Health.

[37] Willis-Shattuck M, Bidwell P, Thomas S, Wyness L, Blaauw D, Ditlopo P (2008). Motivation and retention of health workers in developing countries: a systematic review. BMC Health Serv Res.

[38] Okello DR, Gilson L (2015). Exploring the influence of trust relationships on motivation in the health sector: a systematic review. Hum Resour Health.

[39] Musgrove P (2011). Financial and other rewards for good performance or results: a guided tour of concepts and terms and a short glossary.

[40] Fritsche GB, Soeters R, Meessen B (2014). Performance-based financing toolkit.

